# The authors reply:

**DOI:** 10.1097/PCC.0000000000003439

**Published:** 2024-03-04

**Authors:** Maya Caroline Andre, Jürg Hammer

**Affiliations:** Both authors: Division of Respiratory and Critical Care Medicine, University of Basel Children´s Hospital, Basel, Switzerland.

Thank you for the opportunity to discuss the correspondence (1) about our report on extracorporeal life support in drowned young children with life-threatening hypothermia and out-of-hospital cardiac arrest (OHCA) (2). While extracorporeal membrane oxygenation (ECMO) therapy in children has its merits as a means of bridging refractory cardiac and/or pulmonary failure until recovery or organ transplantation (3), evidence is missing that ECMO therapy—although technically feasible—has merits in the treatment of hypoxia-associated cerebral injury in the young child. Hence, the intention of summarizing the case report literature was to stimulate and encourage a discussion about selecting children with drowning-associated hypothermia who may be eligible for ECMO treatment, but importantly also to point out the reported dismal neurologic outcomes.

First, the decision against ECMO does not mean to renounce performing good quality cardiopulmonary resuscitation (CPR) and active external and internal rewarming. This approach is exemplified by the eight children treated with conventional CPR who were pulseless on arrival and had a mean duration (± sd) of cardiac arrest of 135 minutes (± 119 min); seven of whom had normal outcome.

Second, at the time of writing our report, we purposely chose not to calculate the Hypothermia Outcome Prediction after ECMO (HOPE) score (4) because it had not been validated in children. As requested, we have calculated the HOPE scores for our patient cohort of children younger than 6 years old with a temperature less than 28°C using the publicly available online tool (www.hypothermiascore.org). In the 30 of 44 children, for whom all required information was complete, the median (interquartile range [IQR]) HOPE scores for patients who died vs. patients who survived was: 13% (IQR, 7–34%) vs. 41% (IQR, 29–61%, respectively; *p* ≤ 0.02) (**Fig. 1**). Using the cutoff value of 10% for the HOPE score, the proportion of children estimated to survive but who died (false positives) was 59% and the proportion of patients estimated to die but who survived (false negatives) was 0%. However, the number of young children in our cohort is too small for a meaningful statistical evaluation and the sd overlaps accordingly. We would therefore strongly encourage a validation of the HOPE score in a larger cohort of hypothermic children before assuming that this score will help pediatric intensivists during critical decision-making of ECMO candidacy.

**Figure 1.**
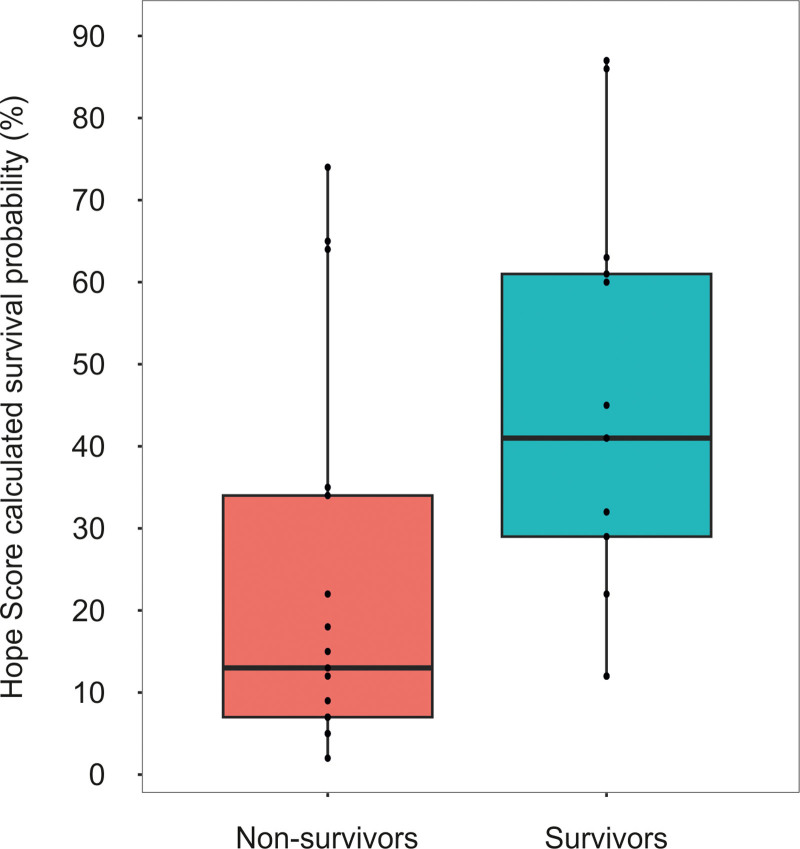
Calculated Hypothermia Outcome Prediction after Extracorporeal membrane oxygenation (HOPE) scores. Survival probabilities in our cohort of extracorporeal membrane oxygenation (ECMO)-treated children younger than 6 yr old with a temperature less than 28°C with out-of-hospital cardiac arrest. The information to calculate the HOPE score was only available for 30 of 44 children treated with ECMO, reported previously (1).

In the context of the above information, we note the lack of scientific evidence to support the notion that extrapolating adult data is the best way to treat young, drowned children with severe hypothermia. In line with this, the 2019 American Heart Association Scientific Statement about pediatric postcardiac arrest care considered the drowning OHCA patient as a population in which there was a “critical knowledge gap” (5). Likewise, uncertainty remains in the “European Resuscitation Council Guidelines 2021: Cardiac arrest in special circumstances” (6) but also in the “European Resuscitation Council Guidelines 2021: Pediatric Life Support” (7).

Last, we agree with the authors of the accompanying correspondence that the internet-based “International Hypothermia Registry” (hypothermia-registry.org) is an excellent platform for increasing the awareness and knowledge of accidental hypothermia in children. All children with drowning-associated hypothermia who are evaluated for ECMO should be reported so that we can acquire evidence-based knowledge to further refine treatment guidelines for young children.

## ACKNOWLEDGMENTS

We would like to thank A. Atkinson, PhD, and C. Sanchez, Pediatric Research Center, University Children’s Hospital Basel, for their advice about the statistical analysis and preparation for the Figure. We also thank D. Trachsel, MD, Division of Respiratory and Critical Care Medicine, University of Basel Children´s Hospital, for critical reading and insightful comments.
